# Association of fasting glucose with lifetime risk of incident heart failure: the Lifetime Risk Pooling Project

**DOI:** 10.1186/s12933-021-01265-y

**Published:** 2021-03-22

**Authors:** Arjun Sinha, Hongyan Ning, Faraz S. Ahmad, Michael P. Bancks, Mercedes R. Carnethon, Matthew J. O’Brien, Norrina B. Allen, John T. Wilkins, Donald M. Lloyd-Jones, Sadiya S. Khan

**Affiliations:** 1 Division of Cardiology, Department of Medicine and Preventive Medicine, Northwestern University Feinberg School of Medicine, 680 N. Lake Shore Drive, 14-002, Chicago, IL 60611 USA; 2 Department of Preventive Medicine, Northwestern University Feinberg School of Medicine, Chicago, IL USA; 3 Department of Epidemiology and Prevention, Wake Forest School of Medicine, Winston-Salem, NC USA; 4 Division of General Internal Medicine and Geriatrics, Department of Medicine, Northwestern University Feinberg School of Medicine, Chicago, IL USA

**Keywords:** Prediabetes, Heart failure, Lifetime risk, Competing risk

## Abstract

**Background:**

Given the rising prevalence of dysglycemia and disparities in heart failure (HF) burden, we determined race- and sex-specific lifetime risk of HF across the spectrum of fasting plasma glucose (FPG).

**Methods:**

Individual-level data from adults without baseline HF was pooled from 6 population-based cohorts. Modified Kaplan–Meier analysis, Cox models adjusted for the competing risk of death, and Irwin’s restricted mean were used to estimate the lifetime risk, adjusted hazard ratio (aHR), and years lived free from HF in middle-aged (40–59 years) and older (60–79 years) adults with FPG < 100 mg/dL, prediabetes (FPG 100–125 mg/dL) and diabetes (FPG ≥ 126 mg/dL or on antihyperglycemic agents) across race-sex groups.

**Results:**

In 40,117 participants with 638,910 person-years of follow-up, 4846 cases of incident HF occurred. The lifetime risk of HF was significantly higher among middle-aged White adults and Black women with prediabetes (range: 6.1% [95% CI 4.8%, 7.4%] to 10.8% [95% CI 8.3%, 13.4%]) compared with normoglycemic adults (range: 3.5% [95% CI 3.0%, 4.1%] to 6.5% [95% CI 4.9%, 8.1%]). Middle-aged Black women with diabetes had the highest lifetime risk (32.4% [95% CI 26.0%, 38.7%]) and aHR (4.0 [95% CI 3.0, 5.4]) for HF across race-sex groups. Middle-aged adults with prediabetes and diabetes lived on average 0.9–1.6 and 4.1–6.0 fewer years free from HF, respectively. Findings were similar in older adults except older Black women with prediabetes did not have a higher lifetime risk of HF.

**Conclusions:**

Prediabetes was associated with higher lifetime risk of HF in middle-aged White adults and Black women, with the association attenuating in older Black women. Black women with diabetes had the highest lifetime risk of HF compared with other race-sex groups.

**Supplementary Information:**

The online version contains supplementary material available at 10.1186/s12933-021-01265-y.

## Introduction

Diabetes is a major risk factor for heart failure (HF) with multiple observational studies demonstrating a two- to fourfold greater risk in middle-aged and older adults [1–6]. In contrast, risk of HF in adults with prediabetes has not been firmly established as some studies have shown an increased risk while other studies in older and Black adults have not [7–12]. A better understanding of HF risk across the spectrum of dysglycemia is needed given the link between the rising prevalence of prediabetes and diabetes and the growing burden of HF. As of 2018, approximately 88 million US adults had prediabetes and 34 million US adults had diabetes[13]; while more than 8 million US adults are expected to develop HF by 2030 [14].

Comprehensive risk assessment that includes lifetime risk and years lived free from HF would enhance communication about the overall risk and impact of prevention to patients, physicians, and public health officials [15, 16]. Furthermore, incorporating the competing risk of death from non-HF causes is essential when estimating lifetime risk of HF because both prediabetes and diabetes are associated with other life-limiting conditions; not accounting for this competing risk results in systematic overestimation of risk [17]. Lifetime risk may also provide a more accurate risk estimate as prediabetes represents an early stage of insulin resistance and clinical cardiac effects may only manifest after a prolonged period of follow-up. Importantly, there are also notable race-sex disparities in HF incidence and outcomes, which may be influenced by diabetes status [18–20]. Age is another important factor in determining risk as evidence suggests that older adults are more likely to revert to normoglycemia [21]. Therefore, a comprehensive assessment of HF risk across the spectrum of dysglycemia stratified by age, sex, and race can help understand potential disparities and inform effective implementation of HF prevention strategies such as aggressive risk factor modification and use of sodium-glucose transporter 2 (SGLT2) inhibitors [22].

We pooled and harmonized data from 6 contemporary population-based cohorts in the Lifetime Risk Pooling Project (LRPP) to estimate: (1) lifetime risks of HF in adults with normal fasting plasma glucose (FPG), prediabetes, and diabetes according to race and sex, after adjusting for the competing risk of non-HF death, (2) years lived free of and with HF in the context of overall survival, and (3) competing adjusted HRs for HF and non-HF death. Analyses were performed in both middle-aged and older adults given the growing prevalence of prediabetes and diabetes with age [13].

## Methods

### Study cohorts

The LRPP dataset has been previously described in detail [23]. Briefly, it consists of pooled individual-level data from multiple population-based cohorts in the US. We included 40,117 White and Black participants free from baseline HF or atherosclerotic cardiovascular disease (CVD) from the following 6 cohorts: Atherosclerosis Risk in Communities Study, Cardiovascular Health Study, Coronary Artery Risk Development in Young Adults Study, Framingham Heart Study starting from 1985, Framingham Offspring Cohort Study starting from 1985, and Multi-Ethnic Study of Atherosclerosis. These cohorts represent contemporary longitudinal studies with available measures of fasting plasma glucose (FPG), other major cardiovascular risk factors, adjudication of HF, and essentially complete follow up for vital status. All data were de-identified, and all study protocols and procedures were approved by the Institutional Review Board at Northwestern University with a waiver for informed consent.

### Demographics and cardiovascular risk factor ascertainment

We categorized participants with baseline FPG levels according to two index age groups—middle age adults between ages 40 and 59 years and older adults between ages 60 and 79 years. We also stratified participants by self-reported race/sex groups for White and Black men and women; we excluded participants of non-Black and non-White self-reported race due to small sample sizes. FPG was measured using validated methods specific to each cohort, as has been previously described [24]. Normal FPG was defined as < 100 mg/dL. Prediabetes was defined as FPG of 100–125 mg/dL and diabetes was defined as FPG of ≥ 126 mg/dL, self-reported physician diagnosis of diabetes or use of anti-hyperglycemic agents. Demographics including age, sex, and race were self-reported. Blood pressure, weight, and height were measured by trained clinical staff and a fasting lipid profile was obtained. The use of medications and smoking status were self-reported. Current smokers were defined as those who had smoked cigarettes within a year of the examination. Hypertension was defined as systolic blood pressure ≥ 140 mmHg, diastolic blood pressure ≥ 90 mmHg, or use of antihypertensive agents. Hyperlipidemia was defined as total cholesterol ≥ 200 mg/dL or use of lipid-lowering agents.

### HF definition and adjudication

Adjudication criteria for incident HF were specific to each cohort and detailed descriptions are provided in Additional file 1: Additional methods. For death events, some cohorts used linkage to the National Death Index to determine the underlying cause of death from death certificate data while other cohorts adjudicated the cause of death by reviewing medical records and autopsy data, when available. Vital status was known for 98% of the pooled cohort.

### Statistical analysis

Lifetime risk of HF was estimated using a modified Kaplan–Meier analysis, which accounts for the competing risk of non-HF related death. In this analysis, fatal events before incident HF were treated as a separate endpoint rather than a censoring event, which reduces overestimation of risk. Lifetime risk of HF for each race-sex group was calculated to age 80 years in the middle-aged index group and to age 90 years in the older index group. We calculated the competing cumulative incidences of HF events compared with non-HF death as the first event during follow-up. The Fine and Gray method was used to estimate the competing hazards for incident HF and non-HF death by FPG status for each race-sex group [25]. Adults with normoglycemia were used as the reference group and the analysis was adjusted for age, BMI, hypertension, smoking status, and hyperlipidemia. The mean survival time or years lived free from HF, with HF, and overall were estimated using the Irwin restricted mean to compare differences in compression of morbidity by FPG status in each race-sex group [16, 26].

All statistical analyses were performed with SAS version 9.2 (SAS Institute) and R version 3.1.2 (The R Foundation).

## Results

### Baseline characteristics

The pooled cohort was stratified by race, sex, and index age. Baseline characteristics of middle-aged (index age 40–59) and older adults (index age 60–79) are described in Table 1 and Additional file 1: Table S1, respectively. A total of 638,910 person-years of follow-up was included in this study. Among middle-aged adults, the prevalence of prediabetes was lowest in White women (20%) and highest in White men (34%). The prevalence of diabetes was higher in Black men (12.0%) and women (11.9%) compared with White men (5.8%) and women (4.3%). Black men and women also had a higher prevalence of hypertension (40% and 44%, respectively) compared with White men and women (24% and 22%, respectively). Similar trends for diabetes and other cardiovascular risk factors were observed in older adults across the race-sex groups.

**Table 1:** Baseline characteristics, fasting plasma glucose categories, and event rates among middle-aged adults (index age 40–59 years)

	Men		Women	
	White n = 6358	Black n = 2191	White n = 7440	Black n = 3181
Mean age, years (SD)	50 (5.6)	49 (5.7)	50 (5.5)	49 (5.6)
Risk factors				
Hypertension, %	24	40	22	44
Mean systolic blood pressure, mmHg (SD)	119 (14.9)	125 (18.7)	115.0 (16.8)	123 (19.5)
Hyperlipidemia, %	56	47	55	49
Mean body mass index, kg/m^2^ (SD)	28 (4.2)	28 (5.6)	27 (5.7)	31 (7.2)
Current smoking, %	24	33	23	23
FPG categories				
Mean glucose, mg/dL (SD)	101 (24.8)	105 (38.8)	96 (25.4)	105 (43.1)
Prediabetes, %	34	28	20	24
Diabetes, %	6	12	4	12
Unadjusted event rates				
HF event/1000PY	4.2	7.2	3.4	6.2
Total death event/1000PY	10.2	14.5	6.7	10.3
Median follow-up time, years	20.9	14.8	21.9	15.8

*HF* heart failure, *SD* standard deviation, *PY* person-year, *FPG* fasting plasma glucose

### Lifetime risk of heart failure by FPG status

During the follow up period, there were a total of 1507 and 3339 cases of incident HF in middle-aged and older adults, respectively. The unadjusted HF event rates were higher in middle-aged Black men and women compared with middle-aged White men and women (Additional file 1: Table S2). As expected, unadjusted HF event rates were higher in older adults compared with middle-aged adults.

**Fig. 1:**
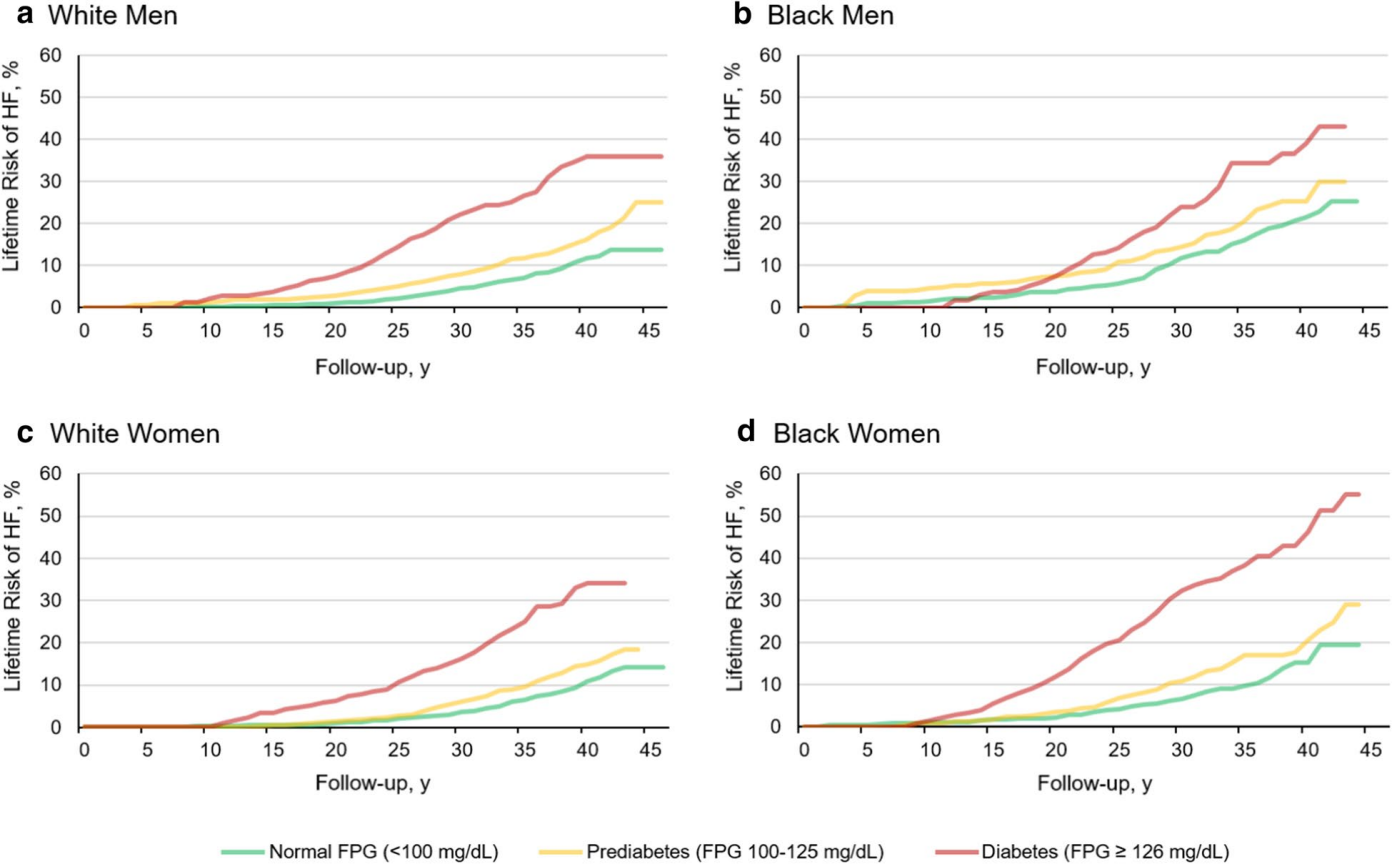
Lifetime risk of heart failure among middle-aged adults (index age 40–59 years) across fasting plasma glucose categories stratified by race and sex. Lifetime risk estimates for heart failure (HF) after adjusting for competing risk of non-heart failure death in middle-aged (index age, 40–59 years) Black and White men (**a**, **b**) and women (**c**, **d**) stratified by fasting plasma glucose (FPG) categories. Lifetime risk for HF was greater in participants with prediabetes or diabetes than those with normal FPG in all groups

The lifetime risk of HF after adjusting for the competing risk of non-HF death stratified by FPG status across race-sex groups is shown in Fig. 1 for middle-aged adults and Additional file 1: Figure S1 for older adults. In middle-aged adults, lifetime risk of HF with prediabetes compared with normal FPG was significantly higher in White men ([7.9%, 95% CI 6.2, 9.7] vs. [4.5%, 95% CI 3.7, 5.3]), White women ([6.1%, 95% CI 4.8, 7.4] vs. [3.5%, 95% CI 3.0, 4.1]), and Black women ([10.8%, 95% CI 8.3, 13.4] vs. [6.5%, 95% CI 4.9, 8.1]). In middle-aged Black men, the lifetime risk with prediabetes compared with normal FPG was not significantly higher ([14.4%, 95% CI 9.6, 19.2] vs. [11.7%, 95% CI 9.1, 14.3]). In older adults, lifetime risk of HF with prediabetes compared with normal FPG was significantly higher in White men ([13.5%, 95% CI 12.0, 14.9] vs. [10.4%, 95% CI 9.2%, 11.6%]) and women ([12.2%, 95% CI 10.8%, 13.6%] vs. [7.8%, 95% CI 6.9%, 8.7%]) but not in Black men ([12.2%, 95% CI 9.0%, 15.3%] vs. [14.6%, 95% CI 11.6%, 17.6%]) or women ([13.5%, 95% CI 10.1%, 16.8%] vs [11.7%, 95% CI 9.4%, 14.0%]). The lifetime risk of HF with diabetes compared with prediabetes or normal FPG was significantly higher in all race-sex groups regardless of age (Additional file 1: Table S3).

When comparing between race-sex groups, the lifetime risk of HF with prediabetes was significantly higher in middle-aged Black men and women compared with middle-aged White men and women, respectively. But there were no significant race-sex disparities in the lifetime risk of HF in older adults with prediabetes. The lifetime risk of HF with diabetes was significantly higher in middle-aged Black women (32.4%, 95% CI 26.0%, 38.7%) than middle-aged White women (16.2%, 95% CI 11.3%, 21.0%). In contrast, the lifetime risk of HF in middle-aged men with diabetes was not different between Black and White men.

### Years lived free of heart failure

**Fig. 2:**
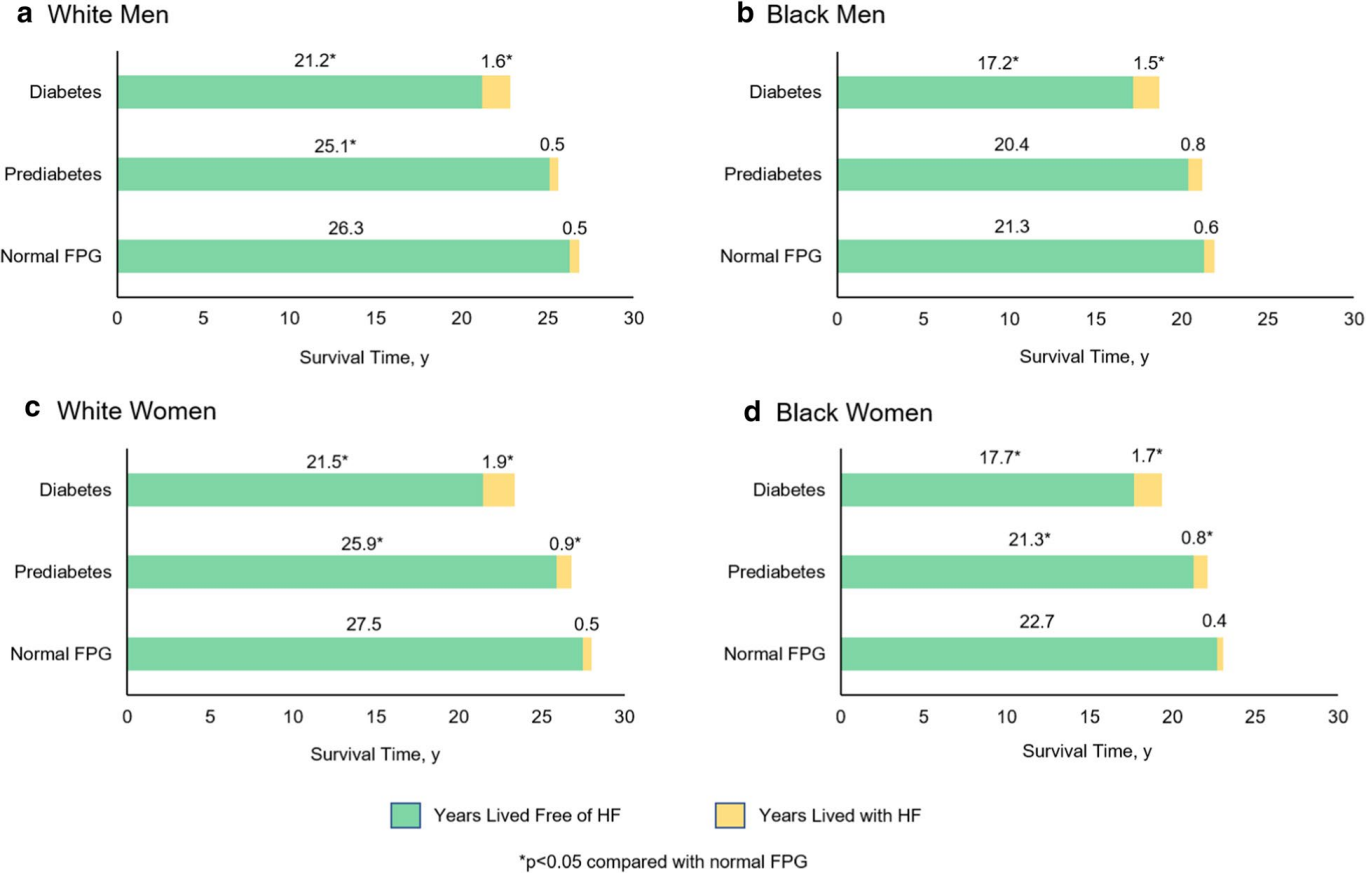
Years lived free from and with heart failure among middle-aged adults (index age 40–59 years) stratified by fasting plasma glucose status. Mean years lived free from and with heart failure (HF) in middle-aged (index age, 40–59 years) Black and White men (**a**, **b**) and women (**c**, **d**) across categories of fasting plasma glucose (FPG). Prediabetes was defined as FPG of 100–125 mg/dL and diabetes defined FPG ≥ 126 mg/dL. Participants with normal FPG (< 100 mg/dL) lived more years free from HF compared with participants with prediabetes or diabetes. Years lived with HF was greater in participants with diabetes than those with normal FPG

Mean years lived free from and with HF for middle-aged adults in each race-sex group stratified by FPG status are provided in Fig. 2 and Additional file 1: Table S4. White men and women with prediabetes lived on average 1.2 and 1.6 fewer years free from HF than White men and women with normal FPG. Black women with prediabetes lived on average 1.4 fewer years free from HF than those with normal FPG. Although not meeting significance, Black men with prediabetes lived on average 0.9 fewer years free from HF than those with normal FPG. In contrast, years lived free from HF were not significantly lower in older adults with prediabetes than normal FPG (Additional file 1: Figure S2 and Table S5). This was the case across all race-sex groups except in White women with prediabetes, who lived on average 0.8 fewer years free from HF than those with normal FPG. Adults with diabetes lived significantly fewer years free from HF, ranging from 4.1 to 6.0 years across race-sex groups, compared with adults with normal FPG. Similar trends were observed in the older adults with diabetes.

### Competing risks of heart failure and non-heart failure mortality by FPG status

We next determined competing hazard ratios for incident HF and non-HF death in adults with prediabetes and diabetes for each race-sex group after adjusting for other risk factors (Table 2). Given that non-HF death encompasses non-HF CVD death and non-CVD death, we provided hazard ratios for both of those competing risks. Adults with normal FPG served as the reference group. In middle-aged adults with prediabetes, the HR of HF was significantly higher in White men (HR 1.39, 95% CI 1.15, 1.69) and women (HR 1.29, 95% CI 1.04, 1.59) as well as Black women (HR 1.59, 95% CI 1.19, 2.13) compared with normal FPG. While older White men and women with prediabetes also had a significantly greater HR of HF, older Black women with prediabetes did not (Additional file 1: Table S6). Black men with prediabetes did not have a significantly greater HR of HF in middle-age (HR 1.15, 95% CI 0.84, 1.57) or older age (HR 0.89, 95% CI 0.65, 1.21). The competing risk-adjusted HR for incident HF in adults with diabetes was significantly higher than those with normal FPG across all race-sex groups. Similar patterns were observed in older adults with diabetes. Finally, we also evaluated the cumulative incidence of HF as the first event (vs. non-HF death). The cumulative incidence of HF was highest in middle-aged Black women with prediabetes (19.2%) and diabetes (44.8%). The cumulative incidence of HF as the first event was greater with worsening dysglycemia across all race-sex groups (Additional file 1: Table S7).

**Table 2:** Adjusted competing hazard ratios for first event (HF event or non-HF death) among middle-aged adults (index age 40–59 years) according to fasting plasma glucose status

	Normal FPG HR (95% CI)	Prediabetes HR (95% CI)	Diabetes HR (95% CI)
White men			
HF	1 [Reference]	1.39 (1.15, 1.69)	2.74 (2.09, 3.59)
Non-HF CVD death	1 [Reference]	1.17 (0.84, 1.64)	1.21 (0.68, 2.14)
Non-CVD death	1 [Reference]	1.13 (0.97 1.32)	1.27 (0.95, 1.68)
Black men			
HF	1 [Reference]	1.15 (0.84, 1.57)	1.68 (1.15, 2.46)
Non-HF CVD death	1 [Reference]	1.26 (0.71, 2.25)	0.94 (0.42, 2.13)
Non-CVD death	1 [Reference]	1.20 (0.91, 1.59)	1.81 (1.28, 2.56)
White women			
HF	1 [Reference]	1.29 (1.04, 1.59)	2.43 (1.79, 3.28)
Non-HF CVD death	1 [Reference]	0.85 (0.46, 1.54)	1.99 (0.94, 4.19)
Non-CVD death	1 [Reference]	1.31 (1.09, 1.58)	2.10 (1.56, 2.82)
Black women			
HF	1 [Reference]	1.59 (1.19, 2.13)	4.00 (2.98, 5.36)
Non-HF CVD death	1 [Reference]	1.75 (0.91, 3.37)	1.40 (0.58, 3.38)
Non-CVD death	1 [Reference]	1.30 (0.98, 1.72)	0.96 (0.65, 1.42)

Fine and Gray method with normoglycemia as reference

Adjusted for age, BMI, HTN, HLD, and smoking

*CVD* cardiovascular disease, *HF* heart failure, *FPG* fasting plasma glucose

## Discussion

In an analysis of 40,117 participants across six cohorts, we quantified lifetime risk of HF in White and Black middle-aged and older adults across the clinical spectrum of glycemia, including prediabetes. We observed (1) higher lifetime risk of HF in middle-aged White adults and Black women with prediabetes but the risk attenuated in older Black women with prediabetes and (2) significant disparities in the risk of HF by race and sex, specifically a disproportionate burden on middle-aged Black women with diabetes. These findings have important implications given the growing prevalence of prediabetes and diabetes as well as a recent rise in HF-related mortality with significant race-sex differences in all three outcomes.

Prior studies have primarily focused on short-term risk of incident HF in adults with prediabetes and have yielded inconsistent results across age and race [7–12]. Our large, pooled cohort allowed us to quantify the association with HF over a longer time horizon and stratify by age, race, and sex. We observed that middle-aged adults with prediabetes had a higher lifetime risk of incident HF and lived fewer years free of HF, on average, than normoglycemic adults. This difference was observed in all race-sex groups except in middle-aged Black men with prediabetes, where the difference did not meet significance, but the trend was similar. Our findings can likely be explained by two, not mutually exclusive, mechanisms. First, cumulative exposure to glucose levels in the prediabetes range during middle-age and beyond may contribute to cardiac dysfunction and development of HF. This explanation is supported by mechanistic and clinical studies demonstrating direct and indirect myocardial effects of insulin resistance and hyperglycemia on myocardial energetics, fibrosis, and subclinical cardiac dysfunction [27–29]. Second, middle-aged adults with prediabetes are more likely to go on to develop diabetes later in life, which results in greater lifetime risk of HF [30]. This contrasts with our findings in older Black women with prediabetes, who did not have a significantly higher lifetime risk of HF. These findings suggest that onset of prediabetes earlier in life is an important HF risk factor in this group that may reflect significant underlying insulin resistance whereas onset of prediabetes later in life may be a more benign manifestation of aging that does not increase the risk of HF in the setting of other competing risks. Further studies are needed to evaluate trends of FPG over time and the association with HF.

These results have significant public health importance given that 88 million US adults in 2018 had prediabetes, with over 63 million of them being under the age of 65 years [13]. Currently the recommendations for therapeutic intervention with metformin in adults with prediabetes are limited to those with additional risk factors (e.g. age less than 60 years, morbid obesity or history of gestational diabetes) [31]. Despite these recommendations, less than 1% of US adults with prediabetes are on metformin [32]. Our results suggest that targeted efforts on HF prevention should be an important aspect of management in adults with prediabetes. Studies are needed to determine if therapies such as SGLT2 inhibitors and intensive blood pressure reduction that have been successful in preventing HF in adults with diabetes will provide a similar benefit in prediabetes [22, 33].

Importantly, our findings can also help convey the importance to patients of early intervention in prediabetes and prevention of diabetes. Both lifetime risk and years lived free from HF are impactful and easy to understand metrics that can help empower patients. It shifts the focus to HF prevention and allows physicians to effectively communicate the benefit of maintaining normal FPG in the context of how many more healthy years the patient may live. Patient and community engagement will be essential in improving healthy life expectancy. Evidence-based lifestyle interventions focused on diet and exercise, such as the Diabetes Prevention Program (DPP) have been shown to reduce the incidence of diabetes in clinical trials and have become the basis of a national partnership of public and private organizations that provide this resource to adults across the country [34, 35]. Expanding access to programs such as DPP with in-person and virtual platforms focused on primordial prevention of diabetes will be key in preventing HF and improving overall cardiovascular health in the US.

Our results also underscore the disparities observed in the context of the relationship between dysglycemia and HF, as middle-aged Black women with diabetes had a relatively higher lifetime risk of HF compared with other groups. There is growing appreciation that this discrepancy is driven, in large part, by underlying social determinants of health, which also contribute to other shared co-morbidities. Black women face racial and gender-based discrimination, socioeconomic adversity, and are at higher risk for developing prediabetes and diabetes after gestational diabetes [36, 37]. These avoidable inequities fuel cardiovascular health disparities by limiting access to health care and creating an environment of chronic mental and physical stress [38]. Underestimation of CVD risk in younger Black women also contributes to disparities, further emphasizing the importance of providing race and sex specific risk estimates [39]. Thus, programs focused on prevention must be sensitive to the barriers and needs within the community and be able to tailor their content and services accordingly. A novel example of this was the blood pressure reduction program focused on Black men throughout barbershops across Los Angeles County [40]. Similar innovative programs are needed to slow the rising burden of HF in Black women with prediabetes and diabetes.

## Study limitations

Our study has potential limitations. A single measure of FPG was used as the primary criteria for determining diabetes status, which may result in misclassification, as participants may change categories of glycemic control during follow-up and this may be differ by race and sex. However, if present this bias would likely result in attenuation of the association and prior investigation has demonstrated good correlation between FPG and hemoglobin A1c in these cohorts [24]. While pooling multiple cohorts provides a larger sample size and improves generalizability, there can be potential for heterogeneity and cohort effects. Although treatment practices and prevalence of prediabetes and diabetes may differ between cohorts and over time, the association with incident HF was similar across cohorts and time periods. The differences between incident HF risk by glycemic status may be driven by other cardiometabolic risk factors such as BMI. Thus, the analyses were adjusted for age, BMI, HTN, HLD, and smoking status.

## Conclusions

We pooled six longitudinal cohort studies to provide race- and sex-specific estimates of the lifetime risk of HF across different categories of FPG in middle-aged and older adults. Our results highlight the role of prediabetes as an independent risk factor for lifetime risk of HF, specifically in middle-aged adults. We also demonstrated important race-sex disparities, as middle-aged Black women with diabetes had the highest lifetime risk, cumulative incidence, and adjusted HR for HF. Given the emergence of novel pharmacological diabetes therapies, such as SGLT2 inhibitors, our findings support future studies examining the benefit of SGLT2 inhibitors earlier in the dysglycemia spectrum for the prevention of HF. Mitigation of lifetime burden of HF will require innovative and culturally tailored prevention programs to target those at greatest risk with impaired FPG.

## Supplementary Information

**Additional file 1.** Additional methods

## Data Availability

The datasets used and/or analyzed during the current study are available from the corresponding author on reasonable request.

## Abbreviations

HF: Heart failure
LRPP: Lifetime Risk Pooling Project
HR: Hazard ratio
CVD: Cardiovascular disease
FPG: Fasting plasma glucose
SGLT2: Sodium-glucose transporter 2
CI: Confidence interval
DPP: Diabetes prevention program
