# Supplementary material for: Association of fasting glucose with lifetime risk of incident heart failure: the Lifetime Risk Pooling Project
Source: Cardiovasc Diabetol. 2021 Mar 22;20:66. doi: 10.1186/s12933-021-01265-y (PMC7983294; doi:10.1186/s12933-021-01265-y)
Supplement: Supplementary file 1 — Additional file 1. Additional methods [file 12933_2021_1265_MOESM1_ESM.docx]

**Additional Material**

**Additional Methods: Cohort Descriptions and Outcome Ascertainment**

Atherosclerosis Risk in Communities Study

The ARIC Study recruited men and women ages 45 to 64 years sampled from 4 US communities with detailed methods previously published.(1) Three methods were used for ascertainment of HF events: 1) participants were contacted annually by phone and records were obtained for any hospitalizations, 2) local hospitals provided lists of hospital discharges with cardiovascular diagnoses, and 3) health department death certificate files were surveyed. Incident HF was defined as the first HF hospitalization or presence of HF code on the death certificate in any position using the *International Classification of Diseases Code, Ninth Revision (ICD-9*) code 428.x and deaths with *ICD-9/10* codes of either 428.x or I50. After excluding individuals due to prevalent CVD or HF and missing covariates, 19,613 individuals were included (345,197 person-years).

Cardiovascular Health Study

The CHS cohort recruited participants from four locations—Forsyth County, North Carolina; Sacramento County, California; Allegheny County, Pennsylvania; and Washington County, Maryland and detailed methods have been previously described(2). CHS participants were classified at baseline according to the presence or absence of preexisting disease for the six cardiovascular outcomes using hospital records and physician confirmation as previously published(3). Ascertainment of HF events and deaths were performed during clinic visits and surveillance calls and records were subsequently obtained. HF was subsequently adjudicated by the CHS Events Subcommittee(4). After excluding individuals due to prevalent CVD or HF and missing covariates, 4,337 individuals were included (57,254 person-years).

Coronary Artery Risk Development in Young Adults Study

The CARDIA study recruited 5115 blacks and whites in 1985-1986 from Birmingham, Alabama; Chicago; Minneapolis; and Oakland, California with full details previously described.(5) All participants were contacted annually by telephone and during scheduled study examinations to report interim hospitalizations. Two members of the endpoint committee adjudicated all hospitalizations and deaths with disagreements resolved by consensus. Hospitalization for HF required both that a final diagnosis of HF had been made by a physician and that medical treatment for HF had been administered during the hospitalization. Death was considered to be due to HF if the adjudicated cause was cardiovascular and if an *International Classification of Diseases, Ninth Revision* (ICD-9) code for HF (428) or cardiomyopathy (425) was noted as a contributory cause. Deaths were reported to the field centers every 6 months, and records were requested after consent had been obtained from the next of kin. After excluding individuals due to prevalent CVD or HF and missing covariates, 4,267 individuals were included (50,109 person-years).

Framingham Heart Study

The Framingham Heart Study started recruiting participants in 1948 and study design and entry criteria have been detailed elsewhere.(6) For the present analysis, we included subjects starting from 1985. Participants have been followed with physical examinations and blood tests every 2 to 4 years. All HF events were reviewed and adjudicated by a panel of three physicians after review of all examinations, as well as physician notes and hospital charts, using previously published criteria.(7) After excluding individuals due to prevalent CVD or HF and missing covariates, 738 individuals were included (9,579 person-years).

Framingham Offspring Study

The Framingham Heart Study offspring cohort started recruiting participants in 1971 and detailed methods have been previously published.(7) For the present analysis, we included subjects starting from 1985. Similar to the parent study, participants have been followed with physical examinations and blood tests every 2 to 4 years. At each visit, HF events were identified, all hospital records reviewed, and diagnosis of HF confirmed by a panel of three physicians.(7) After excluding individuals due to prevalent CVD or HF, and missing covariates, 5,660 individuals were included (95,160 person-years).

Multi-Ethnic Study of Atherosclerosis (MESA)

The MESA cohort recruited asymptomatic participants ages 45 to 84 years from six US communities between 2000-2002, and included white, black, Hispanic, and Asian individuals with detailed methods previously described.(8). All participants were contacted by telephone every 6 to 9 months to inquire about interim hospitalizations and medical records were obtained. HF events were adjudicated by two physicians after review of all available study visits and medical records. Any disagreements were classified by full review committee as previously published.(9) After excluding individuals due to prevalent CVD or HF, and missing covariates, 5,502 individuals were included (61,967 person-years).

**REFERENCES**

1. The Atherosclerosis Risk in Communities (ARIC) Study: design and objectives. The ARIC investigators. Am J Epidemiol 1989;129:687-702.

2. Fried LP, Borhani NO, Enright P et al. The Cardiovascular Health Study: design and rationale. Ann Epidemiol 1991;1:263-76.

3. Psaty BM, Kuller LH, Bild D et al. Methods of assessing prevalent cardiovascular disease in the Cardiovascular Health Study. Ann Epidemiol 1995;5:270-7.

4. Ives DG, Fitzpatrick AL, Bild DE et al. Surveillance and ascertainment of cardiovascular events. The Cardiovascular Health Study. Ann Epidemiol 1995;5:278-85.

5. Friedman GD, Cutter GR, Donahue RP et al. CARDIA: study design, recruitment, and some characteristics of the examined subjects. J Clin Epidemiol 1988;41:1105-16.

6. Dawber TR, Kannel WB, Lyell LP. An approach to longitudinal studies in a community: the Framingham Study. Ann N Y Acad Sci 1963;107:539-56.

7. Kannel WB, Feinleib M, McNamara PM, Garrison RJ, Castelli WP. An investigation of coronary heart disease in families. The Framingham offspring study. Am J Epidemiol 1979;110:281-90.

8. Bild DE, Bluemke DA, Burke GL et al. Multi-Ethnic Study of Atherosclerosis: objectives and design. Am J Epidemiol 2002;156:871-81.

9. Choi EY, Rosen BD, Fernandes VR et al. Prognostic value of myocardial circumferential strain for incident heart failure and cardiovascular events in asymptomatic individuals: the Multi-Ethnic Study of Atherosclerosis. Eur Heart J 2013;34:2354-61.

**Table S1. Baseline Characteristics, Fasting Plasma Glucose Categories, and Event Rates Among Older Adults (Index Age 60-79 years)**

|  | **MEN** | | **WOMEN** | |
| --- | --- | --- | --- | --- |
|  | **White**  **n = 7431** | **Black**  **n = 1806** | **White**  **n = 9198** | **Black**  **n = 2512** |
| Mean Age, years (SD) | 65 (5.1) | 66 (5.3) | 66 (5.3) | 66 (5.4) |
| **Risk Factors** |  |  |  |  |
| Hypertension, % | 47 | 63 | 48 | 68 |
| Mean Systolic Blood Pressure, mm Hg (SD) | 128 (19.0) | 131 (21.0) | 128 (20.3) | 133 (22.2) |
| Hyperlipidemia, % | 55 | 52 | 71 | 65 |
| Mean Body Mass Index, kg/m^2^ (SD) | 28 (5.1) | 28 (5.4) | 27 (5.2) | 30 (6.3) |
| Current Smoking, % | 14 | 21 | 15 | 13 |
| **FPG Categories** |  |  |  |  |
| Mean Glucose, mg/dL (SD) | 108 (31.1) | 111 (39.1) | 103 (28.3) | 113 (47.5) |
| Prediabetes, % | 41 | 33 | 32 | 30 |
| Diabetes, % | 12 | 21 | 9 | 22 |
| **Unadjusted Event Rates** |  |  |  |  |
| HF Event/1000PY | 13.0 | 12.4 | 10.9 | 12.7 |
| Total Death Event/1000PY | 31.0 | 31.7 | 25.2 | 23.4 |
| Median follow-up time, years | 14.1 | 10.8 | 14.7 | 12.8 |

HF: heart failure; SD: standard deviation; PY: person-year; FPG: fasting plasma glucose

**Table S2. Unadjusted Heart Failure Event Rates by Fasting Plasma Glucose Status**

|  | Index Age | Unadjusted Heart Failure Event Rate (per 1000 person-years) | | | |
| --- | --- | --- | --- | --- | --- |
|  |  | White Men | Black Men | White Women | Black Women |
| Normal FPG | Age 40-59 | 2.8 | 5.2 | 2.4 | 3.2 |
|  | Age 60-79 | 10.3 | 10.9 | 8.8 | 9.7 |
| Prediabetes | Age 40-59 | 5.3 | 7.8 | 5.0 | 7.1 |
|  | Age 60-79 | 13.1 | 10.6 | 12.3 | 10.3 |
| Diabetes | Age 40-59 | 14.5 | 16.6 | 13.8 | 22.0 |
|  | Age 60-79 | 25.0 | 19.5 | 21.9 | 24.6 |

FPG: fasting plasma glucose

**Table S3. Lifetime Risk of Heart Failure According to Fasting Plasma Glucose Status**

|  | Index Age | Lifetime Risk % (95% CI), Adjusted for Competing Risks | | | |
| --- | --- | --- | --- | --- | --- |
|  |  | White Men | Black Men | White Women | Black Women |
| Normal FPG | Age 40-59* | 4.5 (3.7, 5.3) | 11.7 (9.1, 14.3) | 3.5 (3.0, 4.1) | 6.5 (4.9, 8.1) |
|  | Age 60-79† | 10.4 (9.2, 11.6) | 14.6 (11.6, 17.6) | 7.8 (6.9, 8.7) | 11.7 (9.4, 14.0) |
| Prediabetes | Age 40-59* | 7.9 (6.2, 9.7) | 14.4 (9.6, 19.2) | 6.1 (4.8, 7.4) | 10.8 (8.3, 13.4) |
|  | Age 60-79† | 13.5 (12.0, 14.9) | 12.2 (9.0, 15.3) | 12.2 (10.8, 13.6) | 13.5 (10.1, 16.8) |
| Diabetes | Age 40-59* | 22.1 (17.1, 27.2) | 23.9 (17.5, 30.4) | 16.2 (11.3, 21.0) | 32.4 (26.0, 38.7) |
|  | Age 60-79† | 27.5 (24.0, 31.0) | 26.5 (20.6, 32.5) | 24.1 (20.6, 27.5) | 31.6 (26.1, 37.1) |

FPG: fasting plasma glucose

*To age 80

†To age 90

**Table S4. Mean Years Lived Free From and With HF in Middle-Aged Adults (Index Age 40-59 years) Stratified by Fasting Plasma Glucose Status**

|  | **Normal FPG (SE)** | **Prediabetes (SE)** | **Diabetes**  **(SE)** |
| --- | --- | --- | --- |
| **White Men** | | | |
| Years free of HF | 26.3 (0.14) | 25.1 (0.18) | 21.2 (0.52) |
| Years with HF | 0.51 (0.05) | 0.50 (0.44) | 1.60 (0.26) |
| Overall survival, years | 26.8 (0.13) | 25.5 (0.41) | 22.8 (0.49) |
| **Black Men** | | | |
| Years free of HF | 21.3 (0.20) | 20.4 (0.30) | 17.2 (0.52) |
| Years with HF | 0.57 (0.08) | 0.84 (0.13) | 1.45 (0.25) |
| Overall survival, years | 21.9 (0.19) | 21.2 (0.27) | 18.6 (0.52) |
| **White Women** | | | |
| Years free of HF | 27.5 (0.09) | 25.9 (0.20) | 21.5 (0.54) |
| Years with HF | 0.49 (0.05) | 0.94 (0.10) | 1.90 (0.30) |
| Overall survival, years | 28.0 (0.08) | 26.9 (0.18) | 23.4 (0.50) |
| **Black Women** | | | |
| Years free of HF | 22.7 (0.13) | 21.3 (0.23) | 17.7 (0.45) |
| Years with HF | 0.39 (0.05) | 0.79 (0.11) | 1.70 (0.21) |
| Overall survival, years | 23.1 (0.12) | 22.1 (0.21) | 19.4 (0.40) |

FPG: fasting plasma glucose; HF: heart failure; SE: standard error

**Table S5. Mean Years Lived Free From and With HF in Older Adults (Index Age 60-79 years) Stratified by Fasting Plasma Glucose Status**

|  | **Normal FPG (SE)** | **Prediabetes (SE)** | **Diabetes**  **(SE)** |
| --- | --- | --- | --- |
| **White Men** | | | |
| Years free of HF | 17.5 (0.14) | 17.2 (0.15) | 13.6 (0.27) |
| Years with HF | 0.75 (0.05) | 0.96 (0.06) | 1.40 (0.13) |
| Overall survival, years | 18.3 (0.13) | 18.1 (0.14) | 15.0 (0.26) |
| **Black Men** | | | |
| Years free of HF | 16.5 (0.33) | 16.6 (0.37) | 14.6 (0.48) |
| Years with HF | 0.80 (0.14) | 0.67 (0.13) | 1.30 (0.27) |
| Overall survival, years | 17.3 (0.33) | 17.3 (0.36) | 15.9 (0.47) |
| **White Women** | | | |
| Years free of HF | 18.8 (0.10) | 18.0 (0.14) | 14.7 (0.30) |
| Years with HF | 0.79 (0.04) | 1.1 (0.07) | 1.60 (0.14) |
| Overall survival, years | 19.6 (0.10) | 19.1 (0.08) | 16.3 (0.28) |
| **Black Women** | | | |
| Years free of HF | 18.5 (0.26) | 18.4 (0.31) | 15.4 (0.40) |
| Years with HF | 0.95 (0.13) | 0.86 (0.13) | 1.60 (0.21) |
| Overall survival, years | 19.5 (0.25) | 19.2 (0.29) | 17.1 (0.38) |

FPG: fasting plasma glucose; HF: heart failure; SE: standard error

**Table S6. Adjusted^*^ Competing Hazard Ratios for First Event (HF event vs. Non-HF death) in Older Adults (Index Age 60-79 years) According to Fasting Plasma Glucose Status**†

|  | **Normal FPG HR (95% CI)** | **Prediabetes HR (95% CI)** | **Diabetes HR (95% CI)** |
| --- | --- | --- | --- |
| **White Men** | | | |
| HF | 1 [Reference] | 1.27 (1.12, 1.43) | 1.85 (1.58, 2.17) |
| Non-HF CVD death | 1 [Reference] | 1.16 (0.94, 1.43) | 1.67 (1.26, 2.20) |
| Non-CVD death | 1 [Reference] | 1.04 (0.94, 1.16) | 1.16 (0.99, 1.35) |
| **Black Men** | | | |
| HF | 1 [Reference] | 0.89 (0.65, 1.21) | 1.36 (0.99, 1.86) |
| Non-HF CVD death | 1 [Reference] | 1.21 (0.77, 1.90) | 1.47 (0.89, 2.43) |
| Non-CVD death | 1 [Reference] | 1.25 (0.99, 1.57) | 0.94 (0.70, 1.27) |
| **White Women** | | | |
| HF | 1 [Reference] | 1.29 (1.15, 1.46) | 1.73 (1.46, 2.05) |
| Non-HF CVD death | 1 [Reference] | 1.03 (0.84, 1.27) | 1.55 (1.17, 2.07) |
| Non-CVD death | 1 [Reference] | 1.15 (1.04, 1.27) | 1.30 (1.10, 1.54) |
| **Black Women** | | | |
| HF | 1 [Reference] | 1.05 (0.81, 1.35) | 2.02 (1.58, 2.58) |
| Non-HF CVD death | 1 [Reference] | 1.72 (1.08, 2.73) | 1.53 (0.91, 2.55) |
| Non-CVD death | 1 [Reference] | 1.23 (0.97, 1.56) | 0.94 (0.70, 1.26) |

^*^Adjusted for age, BMI, HTN, HLD, and smoking

†Fine and Gray Method with no diabetes as reference.

HR: hazard ratio; CVD: cardiovascular disease; HF: heart failure; FPG: fasting plasma glucose

**Table 7. Cumulative Incidence of Heart Failure According to Fasting Plasma Glucose Status**

|  | Index Age | Cumulative Incidence (%), Adjusted for Competing Risk | | | |
| --- | --- | --- | --- | --- | --- |
|  |  | White Men | Black Men | White Women | Black Women |
| Normal FPG | Age 40-59* | 7.4 | 9.3 | 7.1 | 9.9 |
|  | Age 60-79† | 22.1 | 18.9 | 20.3 | 20.0 |
| Prediabetes | Age 40-59* | 12.3 | 13.8 | 12.4 | 19.2 |
|  | Age 60-79† | 27.8 | 19.0 | 25.5 | 25.6 |
| Diabetes | Age 40-59* | 27.1 | 27.6 | 27.8 | 44.8 |
|  | Age 60-79† | 31.4 | 33.4 | 33.2 | 40.0 |

FPG: fasting plasma glucose

*To age 80

†To age 90

**Figure S1. Lifetime Risk of Heart Failure Among Older Adults (Index Age, 60-79 years) Across Fasting Plasma Glucose Categories Stratified by Race and Sex**


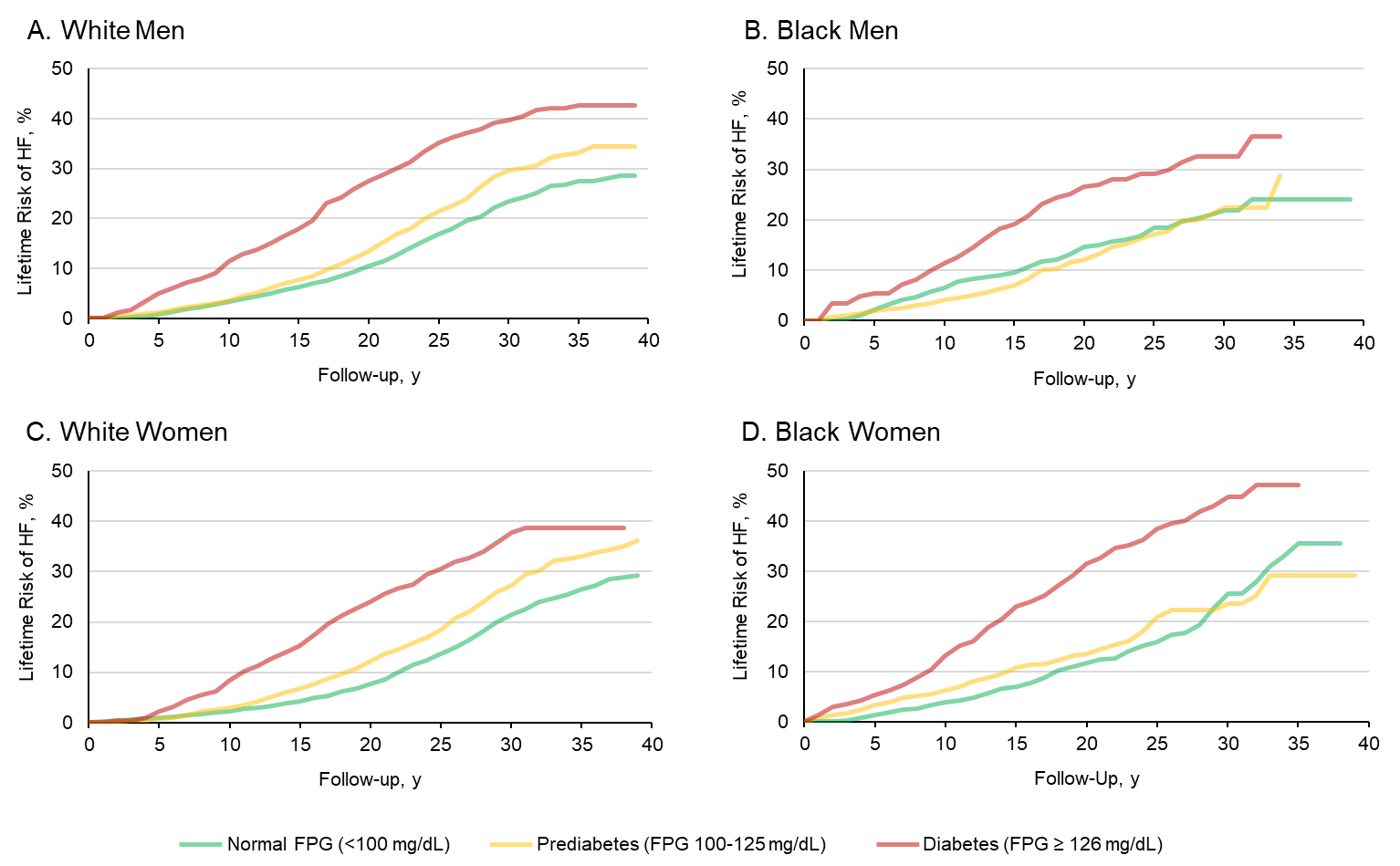


Lifetime risk estimates for heart failure (HF) after adjusting for competing risk of non-heart failure death in older (index age, 60-79 years) black and white men (A, B) and women (C, D) stratified by fasting plasma glucose (FPG) categories. Lifetime risk for HF was greater in participants with diabetes than those with normal FPG in all groups. Lifetime risk for HF in participants with prediabetes was only greater in white adults than those with normal FPG.

**Figure S2. Years Lived Free From and With Heart Failure Among Older Adults (Index Age, 60-79 years) by Fasting Plasma Glucose Categories**

**
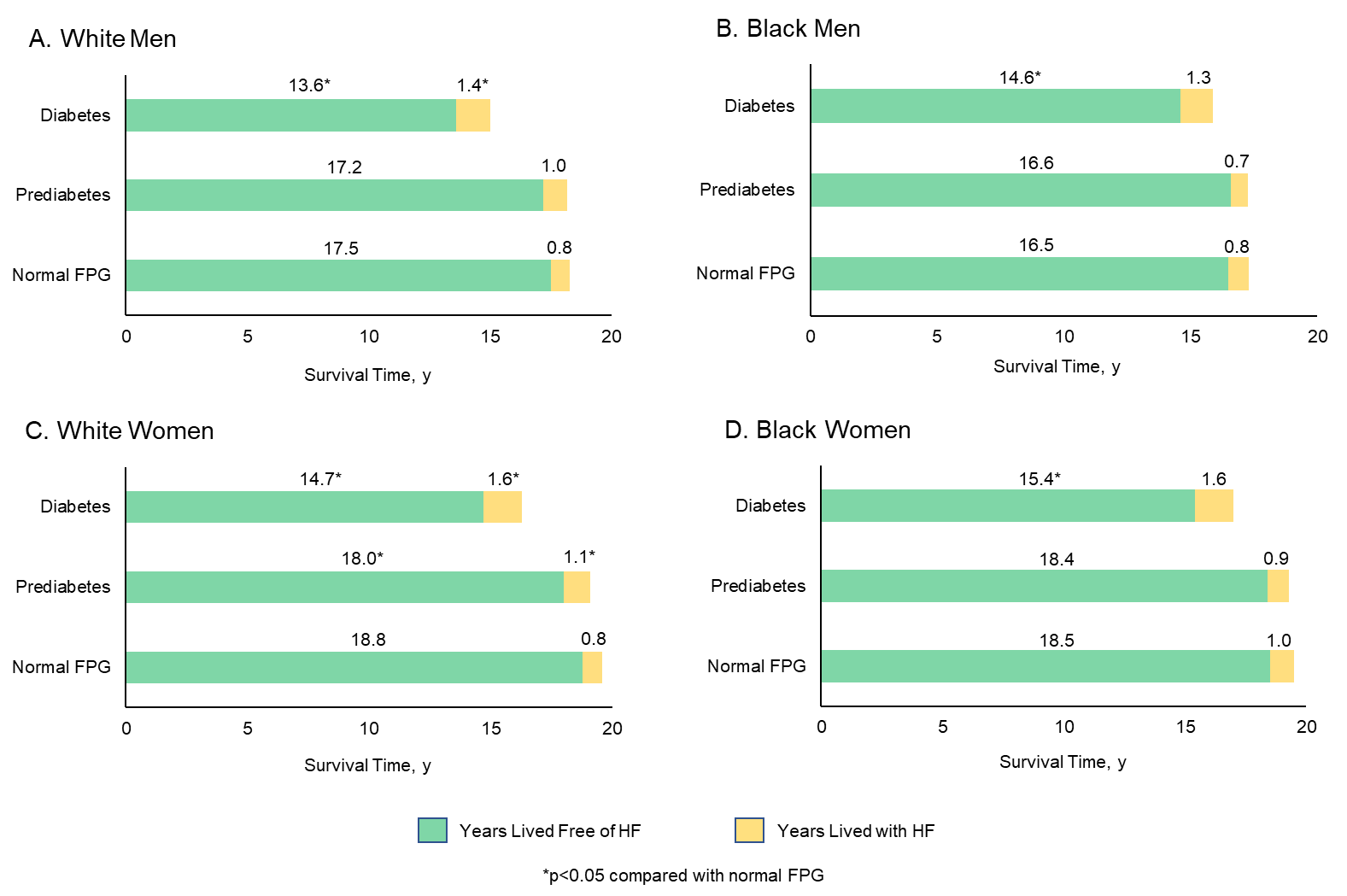
**

Mean years lived free from and with heart failure (HF) in older (index age, 60-79 years) black and white men (A, B) and women (C, D) across categories of fasting plasma glucose (FPG). Prediabetes was defined as FPG of 100-125 mg/dL and diabetes defined FPG ≥126 mg/dL. Participants with normal FPG (<100 mg/dL) lived more years free from HF compared with participants with diabetes. Years lived with HF was greater in white participants with diabetes than those with normal FPG.
